# Knowledge, attitudes and practices of healthcare professionals toward the novel coronavirus during the early stage of COVID-19 in a lower-and-middle income country, Bangladesh

**DOI:** 10.3389/fpubh.2022.988063

**Published:** 2022-09-16

**Authors:** Muhammad Mainuddin Patwary, Md. Riad Hossain, Rabeya Sultana, Ahmad Riaz Dazhamyar, Ali Davod Parsa, Russell Kabir, Sheikh Shoib

**Affiliations:** ^1^Environment and Sustainability Research Initiative, Khulna, Bangladesh; ^2^Environmental Science Discipline, Life Science School, Khulna University, Khulna, Bangladesh; ^3^Institute of Disaster Management, Khulna University of Engineering & Technology, Khulna, Bangladesh; ^4^Mental Health & Disability Officer at Organization for Health Promotion & Management (OHPM), Kabul, Afghanistan; ^5^School of Allied Health, Faculty of Health, Education, Medicine and Social Care, Anglia Ruskin University, Chelmsford, United Kingdom; ^6^Department of Psychiatry, Jawahar Lal Nehru Memorial Hospital, Kashmir, India

**Keywords:** KAP, COVID-19, SARS-CoV-2, healthcare workers, lower-and-middle income country, Bangladesh

## Abstract

Healthcare workers (HCWs) are at an increased risk of COVID-19 infection because of their direct exposure to suspected and confirmed coronavirus patients in healthcare facilities. This condition is even more acute in low-and lower-middle-income countries (LMICs). Given the poor healthcare settings of Bangladesh, it is challenging to halt the spread of infection without proper knowledge, attitudes, and good behavioral practices (KAPs). Therefore, this study conducted a cross-sectional study from May 5 to 31, 2020, with 203 healthcare professionals to determine the knowledge, attitudes, and practices (KAP) toward COVID-19. Participants were doctors, nurses, dentists, and allied health professionals. A self-administered questionnaire including several KAP-related items aligned with the World Health Organization (WHO) guidelines was distributed over various online platforms to collect data. Bivariate and multivariable logistic regression analyses were conducted to determine the factors influencing KAP levels. The majority of participants were male (52.22%). The prevalence of high knowledge levels, positive attitudes, and good practices were 51.23, 45.81, and 49.75%, respectively. Social media was the most common source for seeking coronavirus information. Workers at private institutions were less likely (OR = 0.56, 95% CI = 0.30–0.95, *p* < 0.05) to be knowledgeable than workers at public institutions. Doctors had more positive attitudes than other healthcare professionals. Older participants showed high rates of good behavioral practices (OR = 1.05, 95% CI = 1.06–1.32, *p* < 0.05) than younger ones. Workers at private institutions had a better practice level toward COVID-19 (OR = 2.11, 95% CI = 1.17–3.83) than those at public institutions. These results point to the necessity for proper training programs for medical professionals that help them gain confidence to deliver the correct treatment to their patients and the need to implement preventative steps during pandemics.

## Introduction

The novel coronavirus disease 2019, known as COVID-19, brought an unprecedented risk to public health worldwide. On January 30, 2020, the World Health Organization (WHO) declared COVID-19 a global public health emergency and urged all countries to take coordinated action to halt the spread of the virus (1). Globally, healthcare workers (HCWs) have been playing frontline roles in fighting the COVID-19 pandemic. However, the lack of resources and unavailability of vaccines prevented HCWs from tackling the virus successfully. Governments worldwide have implemented non-pharmaceutical measures, including stopping economic activity, restricting the movement of people, and maintaining social distancing to combat the spread of the infection (2). Despite these measures, the world has witnessed more than 500 million cases and more than six million death as of July 1, 2022 (3).

On March 8, 2020, Bangladesh reported its first case of novel coronavirus and consequently, first death from COVID-19 confirmed on March 18, 2020 (4). As of July 1, 2022, Bangladesh reported COVID-19 cases surpassed 1.9 million, and deaths exceeded 29,000 (5). In an immediate response to halt COVID-19, the Bangladesh government took several steps, including reducing international flights, checking and quarantining incoming travelers for 14 days, declaring the closing of educational institutions, implementing a nationwide lockdown, regulating inter-district movements, suspended commercial activities except for essential services, and ceased social gatherings (2, 6, 7). Further, several organizations voluntarily disseminated extensive public service announcements (PSAs) on COVID-19, stressing the need of public health measures including handwashing, mask use, and social isolation (8). Being one of the world's most populous nations, Bangladesh still experienced significant difficulties keeping track of people's knowledge, attitudes, and behaviors regarding the newly emerged and potentially devastating COVID-19 pandemic (9).

HCWs are at an increased risk of infection because of their direct exposure to suspected and confirmed coronavirus patients in healthcare facilities. This condition is even more acute in low and lower-middle-income countries (LMICs), where health service capacity is poor, and population density is high (10). It has been reported that the increased demand for healthcare services combined with a shortage of qualified medical professionals led to a high rate of COVID-19 infection among HCWs (11). Further, because of long working hours caused by the lack of personnel, HCWs often fail to ensure proper safety, leading to infection (12). A meta-analysis reported that the prevalence rate of HCWs infected with SARS-CoV-2 was 10.1%, including 4.2% in China, 9% in Italy, and 17.8% in the USA (13). In Bangladesh, the mortality rate of HCWs was relatively low (0.05 per 100,000 population); however, these data don't accurately portray the actual scenario since testing capacity and research data were inadequate (14). Studies revealed that HCWs' lack of knowledge and misconceptions may have led to delays in diagnosis and poor infection prevention practices (11, 15). Experience gained from the previous Severe Acute Respiratory Syndrome (SARS) outbreak in 2003 revealed that inadequate knowledge, negative attitudes, and poor behavioral practices (KAPs) regarding infectious diseases impeded containment and inhibited further transmission (16). Thus, HCWs need to be up-to-date about COVID-19 to protect themselves from infection and prevent the spread of the virus within the same hospital (17).

Earlier studies have reported KAPs regarding COVID-19 among HCWs in different contexts (11, 15, 17–19). A study conducted among 686 HCWs in Ethiopia reported that 73.3% of participants had satisfactory knowledge, 54.8% had a good attitude, and 61.5% practiced COVID-19 preventive measures. These participants' knowledge levels were negatively correlated with attitude and practice scores (20). Another study in Turkey found that 91.66% (*n* = 251) HCWs answered correctly for knowledge-based questions, 85.96% (*n* = 251) for precautionary measures questions, and knowledge scores were positively associated with preventive behavior (21). A study in Nepal found that 76% of HCWs reported adequate knowledge, 54.7% reported positive attitudes, and 78.9% reported behavioral practices; here, knowledge was positively associated with attitudes and practices (19). A global systematic review with 20 studies including 12,072 HCWs reported a 75.8% had good knowledge, 74.6% had positive attitudes, and 79.8% had good practices toward COVID-19 (15). Based on this literature and the evolving COVID-19 scenario, it is still necessary to identify the coronavirus's KAP among HCWs. Such findings would give more insight into how Bangladesh might avoid the continued spread of the outbreak.

The healthcare sector, particularly in Bangladesh, is of great concern (10, 22). In the early stages of COVID-19, Bangladesh lacked the necessary healthcare infrastructure, a shortage of personal protective equipment (PPE), and testing kits to effectively contain the spread of the disease (23). As a result, HCWs might have been deprived of systematic training regarding COVID-19 precautionary measures. Further, hospital administration turned away patients without protective gear. These feelings of unequal treatment influenced them to hide their symptoms, which complicated the treatment of regular patients (23). Such poor healthcare services, overcrowded environments in the hospital, and lack of isolation facilities are likely to be compounded by insufficient knowledge and poor infection control and practices mechanism of HCWs that lead to further transmission of infection (24). Like other countries, Bangladesh also followed WHO-recommended guidelines and organized training by government health institutes across the country to prevent the spread of disease (25, 26). Despite the availability of resources, significant numbers of HCWs still had limited knowledge, attitudes, and practices toward COVID-19. Given the limited healthcare settings of Bangladesh, it would be challenging to halt the spread of infection without proper knowledge and good behavioral practices. There is a dearth of studies to date that calculate the KAPs toward COVID-19 among HCWs in Bangladesh.

In the present study, we determined the KAPs regarding COVID-19 among HCWs. In Bangladesh, there have been two studies on the KAP of HCWs. One study conducted the KAPs of COVID-19 during the early stage of the pandemic among 393 healthcare workers (27). However, it focused only on PPE as a means of preventive practices and provided little details about other measures such as knowledge and attitudes. Another study employed a relatively large sample of HCWs but was restricted to a single area of Bangladesh and performed just before the second wave of the pandemic when the pandemic had already attained great attention, and several initiatives had been taken to enhance public awareness (14). Considering this research gap, the present study aimed to determine KAPs toward COVID-19 among HCWs using a nationwide sample during the early stage of the pandemic.

## Methods

### Study design and participants

We conducted a cross-sectional study through online platform. Physicians and other medical staff in Bangladesh who have licenses from the Bangladesh Medical and Dental Council or the Bangladesh Nursing and Midwifery Council were considered for the target participant of this study. The study was conducted between May 5, 2020, and May 31, 2020. The researcher disseminated a semi-structured Google form questionnaire across their own social networks (including private Facebook and WhatsApp groups) to collect data. The survey was undertaken using a convenient sampling strategy. This study was not motivated by a specific hypothesis and was, instead, mostly exploratory. This is why a formal statistical test or power analysis was not used to estimate the minimal sample size in advance. Instead, we calculated after the fact that a sample size of 203 healthcare workers would have allowed us to estimate a two-sided 95% confidence interval with strict precision of 5%, even if only 20% of the study participants could have developed good knowledge, attitude, and good practice toward the COVID-19 pandemic. The study was carried out in accordance with the Declaration of Helsinki and the highest ethical standards were maintained throughout the study. Every participant who took part in the research first gave their electronic permission to take part. Ethical approval for this research was granted by the Institute of Disaster Management at Khulna University of Engineering & Technology, Khulna 9203, Bangladesh.

### Measures

The survey measured sociodemographic characteristics, including gender, age, place of residence, current living status, education, healthcare professions type, frontline status, type of healthcare institution, work experience, and daily working hours. Gender was assessed by asking participants whether they male or female. Age was determined as the two group: ≤ 30 or above 30. Place of residence of the respondents were defined as urban or rural. Respondents were asked about their current living status on three options including living with family members, living with non-family members or living alone. Education level of the participants were classified into five groups: college (2 years of post-secondary education), undergrad (4 years of post-secondary education), graduate (undergrad completed but not enrolled in Masters level education), postgraduate (enroll in or completed master's) and advance (MPhill, PhD) (7, 23). Participants professions were further categorized into four groups: doctors who passed a Bachelor of Medicine and Bachelor of Surgery (MBBS) and practiced medicine; nurses who provided technical assistance to doctors as well as those involved in administrative work at hospitals; dentists who completed a Bachelor of Dental Surgery (BDS) degree and practiced dentistry; and allied health professionals such as physiotherapists, occupational therapists, mental health counselors, and physician assistants (23). Frontline status was determined by asking whether they directly involved with any COVID-19 patient care. Type of healthcare institute was characterized by public vs. private institute. Work experience of the respondents were categorized as <5 years, 5–9 years or >9 years of experience. The daily work hours were determined as <8 h vs. ≥8 h. The KAP assessment was evaluated with WHO guidelines. In addition, participants' sources of COVID-19 related information were evaluated. Options included governmental health agencies, international agencies (e.g., WHO), medical journals, hospital training programs, public and private media, online media, and traditional news sources.

#### Knowledge assessment

Knowledge regarding COVID-19 was assessed using eight questions, including the type of infection, common symptoms, incubation period, mode of transmission, the likelihood of exposure, and effectiveness of the mask. Respondents were asked to rate their responses as “yes,” “no,” or “don't know.” Correct responses were recorded with a score of 1, and an incorrect or uncertain answers received a score of 0 following (28, 29). The total score for knowledge ranged from 0 to 8. Individual knowledge scores were assessed with scores above the mean as good and otherwise as poor knowledge regarding COVID-19. This threshold between good and poor knowledge enhances finding's interpretability and responds to variations in information sources across groups (30). Internal consistency of the knowledge scale was determined using Cronbach's alpha. This score was 0.78, indicating that the data were internally consistent.

#### Attitude assessment

Attitudes toward COVID-19 were assessed by asking five questions: fear of COVID-19 infection, worry about social support, disclosure of patient's exposure to the doctor, willingness to treat COVID-19 patients, and feelings of fatigue after the outbreak. Respondents were asked to rate their attitudes toward each question on a 5-point scale from not at all (0) to very high (4). The overall score was the sum of the five questions and ranged from 0 to 20. Individuals who scored higher than the mean were categorized as having a positive attitude, and those who scored lower than the mean were labeled as having a negative attitude. This classification also corresponds with prior research, aids in findings interpretation, and addresses demographic variations (30). Cronbach's alpha was 0.76, indicating a high degree of internal consistency.

#### Practice assessment

Behavioral preventative measures were assessed with eight items, including maintaining quarantine with family, washing hands, participating in a COVID-19 related training program, using and removal of PPE in the hospital, using of medical mask, and avoiding social gatherings. Each item was answered on a 5-point scale ranging from never (0) to always (4). The total score was the sum of the eight items and ranged from 0 to 32. A score above the mean indicated good practices, and a score below the mean indicated poor practices. Once again, this score was classified into two levels for the same reasons as the knowledge and attitude classifications (30). Cronbach's alpha was 0.79, indicating a high degree of internal consistency.

### Statistical analysis

Frequency distributions were used to assess healthcare professionals' KAPs toward COVID-19 transmission. Categorical data were presented as frequencies (%), while continuous data were displayed as means and standard deviations (SD). We used the Shapiro-Wilk test to check the normality of the data. Since our data failed to meet the assumption of a normal distribution, non-parametric tests were used to determine the relationship between mean KAP scores and sociodemographic variables. Univariate analyses for association between KAP levels and sociodemographic variables were performed using Chi-square or Kruskal-Wallis tests when appropriate. Multivariable logistic regression analysis was conducted to determine predictive factors of KAP levels while holding other sociodemographic factors constant. Only significant variables in the univariate analysis were included in the multivariate analysis. The significance of the associations was determined with odds ratios (OR) and 95% confidence intervals (CI). SPSS statistical software (version 26) was used to analyze the data.

## Results

### Sociodemographic characteristics of the sample

Table 1 summarizes the sociodemographic characteristics of the respondents. About half were female (47.78%, *n* = 97). More than half were 30 years old or younger, and the majority lived in an urban area (95%, *n* = 194). Nearly 85% (*n* = 172) lived with their family members during the pandemic. More than half (52.2%, *n* = 106) received at least a graduate level of education. Regarding profession type, most were doctors (*n* = 150), followed by nurses (*n* = 24), dentists (*n* = 22), and allied health professionals (*n* = 7). Most (75%, *n* = 121) of worked for public hospitals. Approximately 40% (*n* = 81) were frontline workers during the pandemic. More than half (52.22 %, *n* =106) had <5 years of job experience after earning their graduation. Over 80% (*n* =169) worked more than 8 h daily.

**Table 1:** Sociodemographic features of the respondents (*N* = 203).

**Variables**	***N* (%)**
**Gender**	
Male	106 (52.22)
Female	97 (47.78)
**Age (years)**	
≤ 30	113 (55.67)
>30	90 (44.33)
**Place of residence**	
Urban	194 (95.57)
Rural	9 (4.43)
**Living status**	
With family members	172 (84.73)
With non-family members	24 (11.82)
Alone	9 (3.45)
**Education**	
College	6 (2.96)
Undergraduate	15 (7.39)
Graduate	106 (52.22)
Postgraduate	67 (33)
Advanced degree (MPhil, Ph.D.)	9 (4.43)
**Healthcare profession**	
Doctor	150 (49.50)
Nurse	24 (7.92)
Dentist	22 (7.26)
Allied health	7 (2.31)
**Healthcare institution type**	
Public	121 (59.61)
Private	82 (40.39)
**Frontline worker**	
Yes	81 (39.90)
No	122 (60.10)
**Years of employment**	
<5 years	106 (52.22)
5–9 years	46 (22.66)
>9 years	51 (25.12)
**Working hours per day**	
<8 h	34 (16.75)
≥8 h	169 (83.25)

### Knowledge assessment

Table 2 demonstrates the knowledge assessment of healthcare professionals in Bangladesh during the pandemic. The mean score was 7.44 (±0.66). About 51% (*n* = 104) achieved high knowledge scores. Most (99.01%, *n* = 201) had good knowledge of the type of COVID-19 infection. All participants knew that the common symptoms of COVID-19 disease were fever, cough, sore throat, and shortness of breath. Most knew that the incubation period of the onset of the disease was up to 14 days, with a mean of 5 days, and that the disease was transmitted through respiratory droplets such as during coughing and sneezing. All reported that close contact with a confirmed case is a significant risk factor for transmission. Most (93.60%, *n* = 190) had good knowledge of the effectiveness of N-95 masks in reducing infection. Most (98.03%, *n* = 199) also reported that people with chronic diseases and those over 60 years old were at the most risk. More than half (54.68%, *n* = 92) recognized that antiviral drugs could reduce symptoms of COVID-19.

**Table 2:** Knowledge assessment of healthcare professionals during COVID-19.

**Statement**	***N*** **(%)**	
	**Correct**	**Incorrect**
COVID-19 is a viral infection	201 (99.01)	2 (0.99)
Its common symptoms are fever, cough, sore throat, and shortness of breath	203 (100)	0 (0.00)
Its incubation period is up to 14 days with a mean of 5 days	201 (99.01)	2 (0.99)
It is transmitted through respiratory droplets such as cough and sneezing	202 (99.01)	1 (0.99)
Close contact with a confirmed case is a significant risk factor for COVID-19	203 (100)	0 (0.00)
N-95 mask is effective in reducing the spreading of COVID-19	190 (93.60)	13 (6.40)
People with chronic disease and over 60 years are at most risk of COVID-19	199 (98.03)	4 (1.97)
Antiviral drugs can reduce the symptom of COVID-19	111 (54.68)	92 (45.32)
**Knowledge score**		
Mean score (±SD)	7.44 (±0.66)	
High	104 (51.23)	
Low	99 (48.77)	

### Attitude assessment

Table 3 presents the attitude assessment of HCWs in Bangladesh during the COVID-19 pandemic. The mean score was 10.27 (±4.54). More than half of respondents (54.19%, *n* = 110) showed negative attitudes toward COVID-19. One-third (38.42%, *n* = 78) were slightly afraid of becoming infected with COVID-19. Around 19% (*n* = 39) reported that they were high levels of worry about social support, while 31.03% (*n* = 63) reported a little worry about social support. Most (36.95%, *n* = 75) agreed that patients should disclose exposures to doctors. Nearly half (45.32%, *n* = 92) were highly willing to treat COVID-19 patients if they got the opportunity. Only 9.85% (*n* = 20) stated unwillingness to treat any COVID-19 patients. Around 38% (*n* = 78) reported feeling a little fatigue after the outbreak, while 15% (*n* = 32) reported high fatigue after the outbreak.

**Table 3:** Attitude assessment of healthcare professionals during COVID-19.

**Statement**	***N*** **(%)**				
	**Not at all**	**A little bit**	**Moderate**	**High**	**Very high**
Afraid of becoming infected with COVID-19	22 (10.84)	78 (38.42)	52 (25.62)	32 (15.76)	19 (9.36)
Being worried about social support	18 (8.87)	63 (31.03)	55 (27.09)	39 (19.21)	28 (13.79)
Patient should disclose their exposure to the doctor	31 (15.27)	17 (8.37)	37 (18.23)	43 (21.18)	75 (36.95)
Willing to treat COVID-19 patients if get an opportunity	20 (9.85)	30 (14.78)	61 (30.05)	47 (23.15)	45 (22.17)
Feelings of fatigue due to overwork during pandemic	33 (16.26)	78 (38.42)	39 (19.21)	32 (15.76)	21 (10.34)
**Attitude score**					
Mean score (±SD)	10.27 (±4.54)				
Positive	93 (45.81)				
Negative	110 (54.19)				

### Practices assessment

Table 4 shows the behavioral practices of respondents toward COVID-19. The mean score was 20.03 (±7.05). Approximately half of the respondents (49.75%, *n* = 102) showed good behavioral practices toward COVID-19. One-third (32.52%, *n* = 66) always maintained quarantine with their family. More than half (50.25%, *n* = 102) washed their hands more often than before. Only 7.88% (*n* = 16) reported participating in training programs on COVID-19, while 13.79% (*n* = 28) reported participating in online training programs. Although only 30% (*n* =61) of respondents reported frequent use of PPE in hospitals, more than half (59%, *n* =119) reported always removing PPE carefully. Nearly two-thirds (58.62%, *n* = 119) reported that they always used a medical mask, while more than half of respondents (53.20%, *n* = 108) reported avoiding social gatherings when going outside.

**Table 4:** Behavioral practice assessment of healthcare professionals during COVID-19.

**Statement**	***N*** **(%)**				
	**Never**	**Rare**	**Sometimes**	**Often**	**Always**
Maintain quarantine with family	26 (12.81)	14 (6.90)	63 (31.03)	34 (16.75)	66 (32.51)
Wash hands more frequently than before	3 (1.48)	2 (0.99)	49 (24.14)	47 (23.15)	102 (50.25)
Participate in a training program for COVID-19	79 (38.92)	24 (11.82)	52 (25.62)	32 (15.76)	16 (7.88)
Participate in an online training program on COVID-19	64 (31.53)	12 (5.91)	65 (32.02)	34 (16.75)	28 (13.79)
Use PPE in hospital	46 (22.66)	10 (4.93)	53 (26.11)	33 (16.26)	61 (30.05)
Remove PPE carefully	5 (2.46)	5 (2.46)	43 (21.18)	31 (15.27)	119 (58.62)
Use a medical mask when go outside	44 (21.67)	8 (3.94)	40 (19.70)	38 (18.72)	73 (35.96)
Avoid social gathering	3 (1.48)	5 (2.46)	44 (21.67)	43 (21.18)	108 (53.20)
**Practice score**					
Mean score (±SD)	20.03 (±7.05)				
Good	101 (49.75)				
Bad	102 (50.25)				

### Information sources

**Figure 1:**
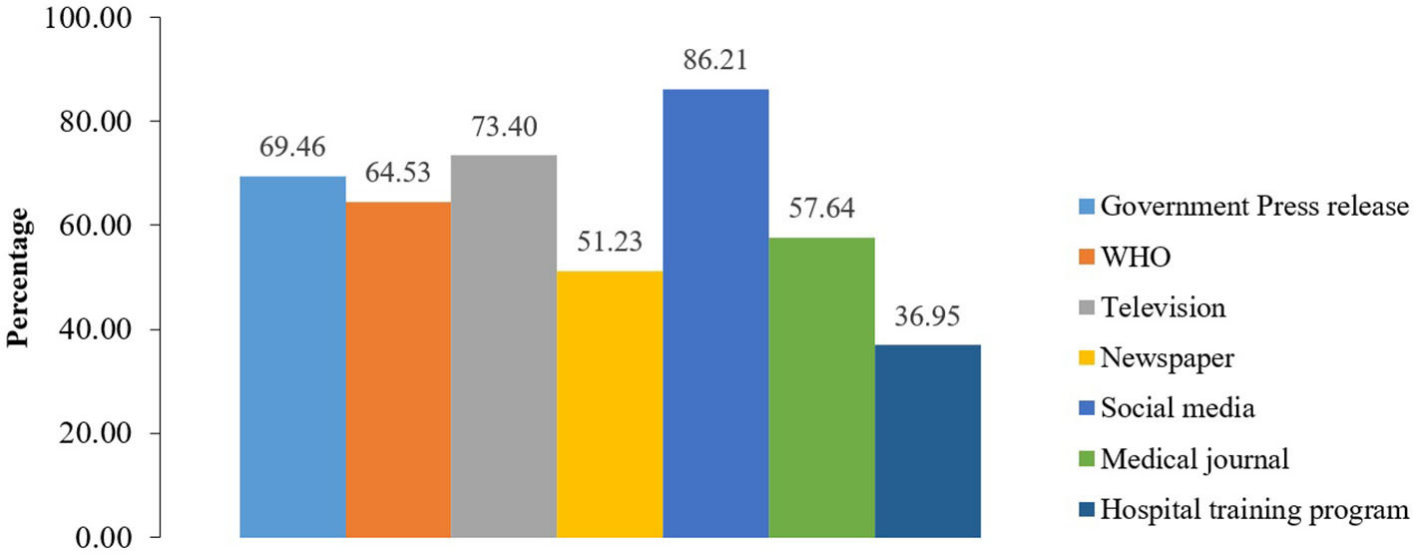
Sources of information on COVID-19 disease among healthcare workers.

Figure 1 illustrates the information sources used by HCWs for COVID-19 information. The majority reported getting information from social media (86.21%). Around 73% of respondents used television for COVID-19 information. About 69% relied on the government press releases for information, while 64% received information from the WHO. More than half used medical journals (57.64%) or newspapers (51.23%) for relevant information. Only 37% used hospital training programs for COVID-19 information.

### Factors influencing the KAPs of HCWs during COVID-19

Table 5 shows the factors influencing the knowledge levels of healthcare professionals during COVID-19. The type of working institution was the only significant factor; respondents working in private institutions were less likely to be knowledgeable than workers in public institutions (OR = 0.56 95% CI = 0.30–0.95, *p* < 0.05).

**Table 5:** Factors influencing knowledge levels regarding COVID-19 among healthcare professionals.

**Variables**	**Knowledge (%)**		**χ^2^ (*p*-value)**	**OR (95% CI)**	***p*-Value**
	**High**	**Low**			
**Gender**					
Male	52 (50)	54 (54.55)	0.42 (>0.05)		
Female	52 (50)	45 (45.45)			
**Age (years)**					
≤ 30	59 (56.73)	54 (54.55)	28.38 (>0.05)		
>30	45 (43.26)	45 (45.45)			
**Place of residence**					
Urban	102 (98.08)	92 (92.93)	3.17 (<0.05)*	Ref.	
Rural	2 (1.92)	7 (7.07)		0.23 (0.04–1.22)	>0.05
**Living status**					
With family members	88 (84.62)	84 (84.85)	1.42 (>0.05)		
With non-family members	11 (10.58)	13 (13.13)			
Alone	5 (4.81)	2 (2.02)			
**Education**					
College	2 (1.92)	4 (4.04)	9.03 (<0.05)*	Ref.	
Undergraduate	3 (2.88)	12 (12.12)		0.63 (0.05–7.2)	>0.05
Graduate	59 (56.73)	47 (47.47)		1.41 (0.17–11.3)	>0.05
Postgraduate	37 (35.58)	30 (30.30)		0.32 (0.07–1.40)	>0.05
Advanced degree	3 (2.88)	6 (6.06)		0.40 (0.09–1.79)	>0.05
**Healthcare profession**					
Doctor	85 (81.73)	65 (65.66)	7.22 (<0.05)*	Ref.	
Nurse	8 (7.69)	16 (16.16)		0.23 (0.05–1.52)	>0.05
Dentist	9 (8.65)	13 (13.13)		0.43 (0.05–3.34)	>0.05
Allied health	2 (1.92)	5 (5.05)		0.45 (0.06–2.97)	>0.05
**Working/study institute**					
Public	69 (66.35)	52 (52.53)	4.04 (<0.05)*	Ref.	
Private	35 (33.65)	47 (47.47)		0.56 (0.30–0.95)	<0.05*
**Frontline role**					
Yes	42 (40.38)	39 (39.39)	0.02 (>0.05)		
No	62 (59.62)	60 (60.61)			
**Years of employment**					
<5 years	60 (57.69)	46 (46.46)	2.68 (>0.05)		
5–9 years	20 (19.23)	26 (26.26)			
>9 years	24 (23.08)	27 (27.27)			
**Working hours**					
<8 h/day	19 (18.27)	15 (15.15)	0.35 (>0.05)		
≥8 h/day	85 (81.73)	84 (84.85)			

*Significant at the 0.05 level (2-tailed).

Table 6 shows the factors influencing the attitude levels of healthcare professionals during COVID-19. Results showed that type of healthcare profession was the only significant factor: doctors were more likely to have positive attitudes than other working professions.

**Table 6:** Factors influencing the attitudes toward COVID-19 among healthcare professionals.

**Variables**	**Attitude (%)**		**χ^2^ (*p*-value)**	**OR (95% CI)**	***p*-Value**
	**Positive**	**Negative**			
**Gender**					
Male	49 (52.69)	56 (51.38)	0.01 (>0.05)		
Female	44 (47.31)	53 (48.62)			
**Age (years)**					
≤ 30	54 (58.06)	58 (53.21)	27.84 (>0.05)		
>30	39 (41.94)	51 (46.79)			
**Place of residence**					
Urban	89 (95.70)	104 (95.41)	0.01 (<0.05)*	Ref.	
Rural	4 (4.30)	5 (4.59)		1.10 (0.28–4.32)	>0.05
**Living status**					
With family members	83 (89.25)	88 (80.73)	3.11 (<0.05)*	Ref.	
With non-family members	7 (7.53)	17 (15.60)		0.31 (0.03–2.89)	>0.05
Alone	3 (3.23)	4 (3.67)		0.68 (0.06–7.35)	>0.05
**Education**					
College	4 (4.30)	2 (1.83)	2.76 (>0.05)		
Undergraduate	9 (9.68)	6 (5.50)			
Graduate	45 (48.39)	60 (55.05)			
Postgraduate	31 (33.33)	36 (33.03)			
Advanced degree	4 (4.30)	5 (4.59)			
**Healthcare profession**					
Doctor	62 (66.67)	87 (79.82)	12.39 (<0.01)**	Ref.	
Nurse	15 (16.13)	9 (8.26)		0.02 (0.00–0.13)	<0.001***
Dentist	9 (9.68)	13 (11.93)		0.02 (0.00–0.15)	<0.001***
Allied health	7 (7.53)	0 (0.00)		0.02 (0.00–0.14)	<0.001***
**Working/study institute**					
Public	51 (54.84)	70 (64.22)	1.60 (>0.05)		
Private	42 (45.16)	39 (35.78)			
**Frontline role**					
Yes	39 (41.94)	42 (38.53)	0.29 (>0.05)		
No	54 (58.06)	67 (61.47)			
**Years of employment**					
<5 years	51 (54.84)	54 (49.54)	1.19 (>0.05)		
5–9 years	22 (23.66)	24 (22.02)			
>9 years	20 (21.51)	31 (28.44)			
**Working hours**					
<8 h/day	16 (17.20)	18 (16.51)	0.02 (>0.05)		
≥8 h/day	77 (82.80)	91 (83.49)			

***Significant at the 0.001 level (2-tailed).

**Significant at the 0.01 level (2-tailed).

*Significant at the 0.05 level (2-tailed).

Table 7 presents the factors influencing the behavioral practices of healthcare professionals during COVID-19. Results showed that age and type of working institution were significant. Participants over 30 years old were more likely to show good behavioral practice than younger participants (OR = 1.05, 95% CI = 1.06–1.32, *p* < 0.05). Participants in private institutions also had high likelihoods of good practices (OR = 2.11, 95% CI = 1.17–3.83).

**Table 7:** Factors influencing behavioral practices regarding COVID-19 among healthcare professionals.

**Variables**	**Practice (%)**		**χ^2^ (*p*-value)**	**OR (95% CI)**	***p*-Value**
	**Good**	**Bad**			
**Gender**					
Male	49 (48.51)	57 (55.88)	1.10 (>0.05)		
Female	52 (51.49)	45 (44.12)			
**Age (years)**					
≤ 30	57 (56.44)	57 (55.88)	43.78 (<0.05)*	Ref.	
>30	44 (43.56)	45 (44.12)		1.05 (1.06–1.32)	<0.05*
**Place of residence**					
Urban	94 (93.07)	100 (98.04)	2.95 (<0.05)*	Ref.	>0.05
Rural	7 (6.93)	2 (1.96)		3.57 (0.68–18.60)	
**Living status**					
With family members	92 (91.09)	80 (78.43)	7.07 (<0.05)*	Ref.	
With non-family members	8 (7.92)	16 (15.69)		0.14 (0.01–0.98)	>0.05
Alone	1 (0.99)	6 (5.88)		0.38 (0.03–3.96)	>0.05
**Education**					
College	4 (3.96)	2 (1.96)	1.05 (>0.05)		
Undergraduate	7 (6.93)	8 (7.84)			
Graduate	51 (50.50)	55 (53.92)			
Postgraduate	34 (33.66)	33 (32.35)			
Advanced degree	5 (4.95)	4 (3.92)			
**Healthcare profession**					
Doctor	75 (74.26)	75 (73.53)	1.62 (>0.05)		
Nurse	11 (10.89)	13 (12.75)			
Dentist	10 (9.90)	12 (11.76)			
Allied health	5 (4.95)	2 (1.96)			
**Working/study institute**					
Public	51 (50.50)	70 (68.63)	6.93 (<0.01)**	Ref.	<0.01**
Private	50 (49.50)	32 (31.37)		2.11 (1.17–3.83)	
**Frontline role**					
Yes	44 (43.56)	37 (36.27)	1.12 (>0.05)		
No	57 (56.44)	65 (63.73)			
**Years of employment**					
<5 years	57 (56.44)	49 (48.04)	1.55 (>0.05)		
5–9 years	20 (19.80)	26 (25.49)			
>9 years	24 (23.76)	27 (26.47)			
**Working hours**					
<8 h/day	15 (14.85)	19 (18.63)	0.51 (>0.05)		
≥8 h/day	86 (85.15)	83 (81.37)			

^**^Significant at the 0.01 level (2-tailed).

^*^Significant at the 0.05 level (2-tailed).

## Discussion

### Summary of the main findings

Globally, healthcare workers are at an increased risk of COVID-19 infection because of their direct exposure to suspected and confirmed coronavirus patients (31). In some cases, healthcare workers (HCWs) were also sources of community transmission. Evidence suggests that overcrowding, lack of proper safety equipment, and absence of isolation facilities are associated with the transmission of disease among HCWs (32). This scenario is likely compounded when HCWs lack awareness of infection and prevention practices. Previous studies show that adequate knowledge, positive attitudes, and good behavioral practices help reduce the risk of infection (33). To the best of our knowledge, this is the first study of its kind in Bangladesh that assesses the KAPs level of HCWs toward COVID-19. Our study found that more than half of HCWs had high levels of knowledge, nearly half showed positive attitudes, and half showed good behavioral practices toward COVID-19.

Regarding our findings on knowledge levels, this parallels other research, such as a previous study with 304 healthcare workers in Pakistan, where half of HCWs had high levels of knowledge regarding COVID-19 (34). Another study in Addis Ababa, Ethiopia, found half of hospital and community pharmacists had high levels of knowledge regarding COVID-19 (35). Our sample finding was slightly lower than other studies. One possible explanation is that the pandemic had already achieved tremendous prominence at the time of this study, and many steps had been made to raise awareness levels among individuals at the time of these studies, so they may have already reached this high level of knowledge on COVID-19. Further, our study reported that most HCWs relied on social media for COVID-19 information, which could have affected their decision-making ability; social media has long been acknowledged to spread false health information (36, 37). Our findings reveal that most respondents correctly answered most of the knowledge-related questions. These findings were in accord with the previously published literature (34). Still, nearly half of our respondents gave incorrect answers on whether antiviral drugs could reduce the symptoms of COVID-19. This finding underscores the necessity for health authorities to continue encouraging HCWs to obtain information from trustworthy sources and engage in training that stress COVID-19's less common presentation and treatment (18).

Less than half of the HCWs showed positive attitudes toward COVID-19. This finding parallels those observed in Pakistan, where only 44% of HCWs reported positive attitudes toward COVID-19 (38). Similarly, some previous studies revealed lower levels of a positive attitude toward infectious diseases (39). In our research, most HCWs were moderate to highly worried about social support and afraid of infecting their family members. This fear could induce negative attitudes toward COVID-19 risk. Earlier studies found that most HCWs were worried of infecting their family members and relatives (40, 41). A study found a similar result where 88% of respondents feared infecting their family, friends, and society (19). Our low proportion of positive attitudes toward COVID-19 might be due to differences in country settings, as Bangladesh has a poor healthcare system that worsened COVID-19 control strategies. The low capacity of the healthcare system in Bangladesh also intensified the fear of infection among HCWs. Doctor-patient ratios in Bangladesh are 5.81 per capita, the second-lowest in South Asia (42). There is not a single critical care bed per 10,000 people in Bangladesh (43). Such an inadequate resources put additional strains on HCWs who were already working in a stressed environment (42). There were also incidents of COVID-19 patients running away from hospitals due to fear of being isolated from their families (44), which may have put additional pressure on HCWs. HCWs endured societal shame, hatred, addressed as virus carriers, and other types of social discrimination during the early phases of the COVID-19 epidemic (45) that likely contributed to negative attitudes toward COVID-19.

Nearly half of the participants showed good precautionary behaviors toward COVID-19. A similar finding in Pakistan reported that 58.9% of HCWs had good practices toward COVID-19 (34). Another study in Bangladesh in early 2021 reported that 62% of HCWs had good preventive practices (14). Our study finding was much lower than some studies, where 78.9% were reported in Nepal (19), 88.7% were reported in Pakistan (17), and 89.7% were reported in China (33). This might be attributed to the lack of good knowledge among HCWs about COVID-19. Having adequate knowledge of the COVID-19 infection could help to adopt good preventive practices. Earlier studies also showed that respondents with good knowledge levels exhibited optimistic attitudes and proactive practices toward COVID-19 (46, 47). More specifically, several malpractices such as not maintaining proper quarantine after work, using face masks more than once, not wearing PPE and N-95 mask regularly while contacting patients, and lack of appropriate training on COVID-19 precautionary practices could be contributed to poor preventive practices during COVID-19 (14). Among various preventive practices in our study, the most frequent were removing PPE carefully regularly (58.62%), followed by avoiding social gatherings (53.20%) and washing hands more frequently than before (50.25%). This finding contrasts with a recent study that found that 80% of HCWs avoided social gatherings during COVID-19. This may be because the HCWs were occupied with COVID-19 patients, which limited their ability to remove PPE frequently. In addition, frontline health care workers in LMICs, particularly in Bangladesh, were required to work in congested workplaces and with inadequate infection prevention and control mechanisms, making it impossible to avoid gathering regularly (24). Jawed et al. (34) also found handwashing to be the most popular practice, and 70% of respondents followed proper hand hygiene, which was higher than in our study. This discrepancy may be because of inadequate water infrastructure and poor infection and prevention control practices (IPC) in hospital settings. A national survey conducted in 2014 in Bangladesh reported that only 2% of HCWs were compliant with the recommended hand hygiene practice due to lack of inadequate infrastructure and poor IPC training (48). Similar problems have been faced by many healthcare facilities in LMICs; for example, one study found that 50% of healthcare facilities in LMICs lacked piped water and 39% lacked handwashing soap (49). Unfortunately, only 30%−35% of respondents of our study maintained quarantine with family, and used a mask while going outside. This might be due to the HCW's fear of infecting their family members and relatives. Further, nearly 23% of HCWs reported that they never used PPE at the hospital during pandemic period. This case occurred during the early stages of the pandemic in Bangladesh, when healthcare workers were unaware of the outbreak. In addition, 40% of the HCWs in our study were from private institutions where COVID-19 patients were not initially treated during the outbreak. This is the result of a shortage of personal protective equipment (PPE) and its questionable quality, which makes it difficult for health care workers (HCWs) to continue their duties during the early stages of the COVID-19 outbreak in Bangladesh (2). Both public and private hospitals in Bangladesh were failed to provide PPE to frontline healthcare workers (50). Such a scenario might have led to substandard PPE use among healthcare workers. It is alarming that few of our participants attended training programs on precautionary practices during COVID-19. Earlier studies indicated that training programs organized by hospitals had an important influence on the prevention of infectious disease outbreaks (51, 52). It is crucial to provide healthcare professionals with the resources they need to develop and use evidence-based knowledge, which could be achieved through proper training (53).

The major sources of information regarding the COVID-19 disease were social media, followed by television, governmental press releases, the WHO website, medical journals, newspapers, and hospital training programs. This finding is in line with a previous study measuring public knowledge of COVID-19 transmission in Pakistan, which found that social media was the top source for seeking coronavirus information, followed by television (54). Another study reported that the HCWs in Ho Chi Minh City used social media as the top source for coronavirus information (55). In contrast, a study in Pakistan found that television, radio, and newspaper were the prevailing sources of coronavirus information among HCWs (34). The reason for using social media as the most common source during COVID-19 could be its easy access and user-friendly features. The Bangladesh government, being aware of the critical need for timely and accurate risk communication during the pandemic, emphasized e-government and social media as means of disseminating information to the public, which could also have contributed to HCW's reliance on social media (2).

Our study confirmed that age, working institution, and healthcare profession were predictive of KAP levels among HCWs in Bangladesh. HCWs in private institutions showed lower knowledge levels than those in public institutions. Similar findings were observed in a recent study in Eastern Ethiopia, where HCWs of public health facilities reported sufficient knowledge levels about COVID-19 (20). The reasons for low knowledge level among private hospital HCWs might be the lack of proper training and inadequate supply of PPE during the early stage of the pandemic. As a result of uncertainty about PPE availability in health care institutions, HCWs in private medical facilities were required to purchase their PPEs. In addition, HCWs in private hospitals were less interested in COVID-19 although they were in direct contact with COVID-19 positive patients (56).

Respondents' type of healthcare profession was another significant factor influencing attitude levels toward COVID-19. Our study found that respondentss who worked as doctors had better attitudes than other professionals. A similar finding was observed in Saudi Arabia, where physicians had a more favorable attitude toward COVID-19 than nurses (57). Another study in Pakistan reported higher attitude scores among physicians than pharmacists and nurses (58). Further, a study in Pakistan found that good knowledge levels were determinants of good attitude levels among community HCWs (38). This might be due to knowledge differences among professionals: the more knowledge, the more positive attitude toward the disease. A good level of knowledge is necessary for creating preventative beliefs, generating good attitudes, and fostering positive behaviors toward disease (59). Doctors also had to work directly with COVID-19 patients and therefore required higher knowledge levels than other professionals. A recent study confirmed that physicians had the highest knowledge scores regarding COVID-19 (57).

Our findings also showed that respondents over 30 had better practices toward COVID-19. Similar results were reported in Eastern Ethiopia, where older HCWs tended to be more cautious than their younger counterparts (20). A systematic review and meta-analysis also indicated that older HCWs had better COVID-19 practices (15). This might be because older HCWs perceived themselves as riskier than younger ones (20). Further, older healthcare workers might have experienced previous pandemics like SARS, H1N1, and MERS and were already alerted to the need for self-protection, cleanliness, and quarantine, so they exhibited better practices toward COVID-19 (60).

In our study, HCWs in private hospitals showed better practices than those in public hospitals. Our findings contradict a previous study that showed HCWs in public hospitals had better preventive behavior scores (21). Perhaps this difference is due to private hospitals in Bangladesh initially refusing to treat patients with COVID-19, giving their HCWs a better chance to strictly adhere to preventative practices. In contrast, public hospitals in Bangladesh had an overcrowded environment that made it challenging to practice acceptable preventive practices (50).

### Limitations

This study has some limitations that should be acknowledged. We included a modest sample size, so our results may not be generalizable to all HCWs in the country. A further drawback is that the questionnaire was developed in a rapid response to the emerging pandemic before the virus spread widely throughout the country. The reliability of our findings depended on the accuracy and recall of the participants. In addition, the sample may have been biased toward certain respondents who could access the internet. This study was undertaken during the early phases of the COVID-19 outbreak in Bangladesh, and it lacked longitudinal follow-up data. Finally, given the cross-sectional nature of this study, we cannot confidently say the observed associations represented cause-and-effect relationships.

## Conclusion

The current study determined the knowledge, attitude, and practice (KAP) levels among HCWs in Bangladesh toward the coronavirus during the early stage of the pandemic. We found more than half of HCWs had a high level of knowledge, nearly half showed positive attitudes, and half reported good behavioral practices. Age, working institution, and type of healthcare profession were determinants of KAPs levels. This study demonstrated a need for an immediate upgradation of the KAP level of HCWs in Bangladesh. It remains crucial to monitor preventative practices and respond accordingly during the pandemic. More in-depth studies are needed to explore the best strategies for combating COVID-19. Finally, a proper training program with incentives for healthcare practitioners could help them build their confidence to provide the right care to their patients and spread awareness among the public about the risks posed by the illness and the need for preventive measures.

## Data availability statement

The raw data supporting the conclusions of this article will be made available by the authors, without undue reservation.

## Ethics statement

The studies involving human participants were reviewed and approved by the research ethical clearance board of the Institute of Disaster Management, Khulna University of Engineering & Technology, Khulna, Bangladesh. The patients/participants provided their written informed consent to participate in this study.

## Author contributions

MP: conceptualization, data curation, methodology, formal analysis, writing—original draft, review and editing. MH: conceptualization, data curation, investigation, writing—review and editing. RS: conceptualization, data curation, writing—review and editing. AD, AP, RK, and SS: writing—review and editing. All authors contributed to the article and approved the submitted version.

## Conflict of interest

The authors declare that the research was conducted in the absence of any commercial or financial relationships that could be construed as a potential conflict of interest.

## Publisher's note

All claims expressed in this article are solely those of the authors and do not necessarily represent those of their affiliated organizations, or those of the publisher, the editors and the reviewers. Any product that may be evaluated in this article, or claim that may be made by its manufacturer, is not guaranteed or endorsed by the publisher.
